# The Role of Sugars in the Regulation of the Level of Endogenous Signaling Molecules during Defense Response of Yellow Lupine to *Fusarium oxysporum*

**DOI:** 10.3390/ijms21114133

**Published:** 2020-06-10

**Authors:** Magda Formela-Luboińska, Tamara Chadzinikolau, Kinga Drzewiecka, Henryk Jeleń, Jan Bocianowski, Jacek Kęsy, Mateusz Labudda, Philippe Jeandet, Iwona Morkunas

**Affiliations:** 1 Department of Plant Physiology, Poznań University of Life Sciences, Wołyńska 35, 60-637 Poznań, Poland; magda.formela-luboinska@up.poznan.pl (M.F.-L.); tamara.chadzinikolau@up.poznan.pl (T.C.); 2 Department of Chemistry, Poznań University of Life Sciences, Wojska Polskiego 75, 60-625 Poznań, Poland; kinga.drzewiecka@up.poznan.pl; 3 Institute of Plant Products Technology, Poznań University of Life Sciences, Wojska Polskiego 31, 60-624 Poznań, Poland; henrykj@up.poznan.pl; 4 Department of Mathematical and Statistical Methods, Poznań University of Life Sciences, Wojska Polskiego 28, 60-637 Poznań; jan.bocianowski@up.poznan.pl; 5 Department of Plant Physiology and Biotechnology, Nicolaus Copernicus University in Toruń, Lwowska 1, 87-100 Toruń, Poland; Jacek.Kesy@umk.pl; 6 Department of Biochemistry and Microbiology, Institute of Biology, Warsaw University of Life Sciences-SGGW, Nowoursynowska 159, 02-776 Warsaw, Poland; mateusz_labudda@sggw.edu.pl; 7 Research Unit “Induced Resistance and Plant Bioprotection”, UPRES EA 4707, Department of Biology and Biochemistry, Faculty of Sciences, University of Reims, P.O. Box 1039, CEDEX 02, 51687 Reims, France; philippe.jeandet@univ-reims.fr

**Keywords:** abscisic acid, benzoic acid 2-hydroxylase, ethylene, Fusarium wilt, hydrogen peroxide, *Lupinus luteus*, phenylalanine ammonia-lyase, sugars, salicylic acid, superoxide dismutase

## Abstract

Soluble sugars such as sucrose, glucose and fructose in plant host cells not only play the role as donors of carbon skeletons, but they may also induce metabolic signals influencing the expression of defense genes. These metabolites function in a complex network with many bioactive molecules, which independently or in dialogue, induce successive defense mechanisms. The aim of this study was to determine the involvement of sucrose and monosaccharides as signaling molecules in the regulation of the levels of phytohormones and hydrogen peroxide participating in the defense responses of *Lupinus luteus* L. to a hemibiotrophic fungus *Fusarium oxysporum* Schlecht f. sp. *lupini*. A positive correlation between the level of sugars and postinfection accumulation of salicylic acid and its glucoside, as well as abscisic acid, was noted. The stimulatory effect of sugars on the production of ethylene was also reported. The protective role of soluble sugars in embryo axes of yellow lupine was seen in the limited development of infection and fusariosis. These results provide evidence for the enhanced generation of signaling molecules both by sugar alone as well as during the crosstalk between sugars and infection caused by *F. oxysporum*. However, a considerable postinfection increase in the level of these signaling molecules under the influence of sugars was recorded. The duration of the postinfection generation of these molecules in yellow lupine was also variable.

## 1. Introduction

Soluble sugars such as sucrose, glucose and fructose in plant host cells not only play the role of donors of carbon skeletons and respiratory substrates, but they may also induce metabolic signals influencing the expression of many genes, including defense genes [1,2,3,4,5,6]. It has been documented that the presence of sucrose enables the plants to acquire an ability to activate efficient defense mechanisms against pathogenic fungi, including systemic fungal pathogens such as *Fusarium oxysporum* Schlecht [4,7,8,9,10,11]. Additionally, trehalose has also been proposed as a signaling molecule causing induction of defense responses in plant hosts against an obligate biotrophic pathogen [12]. Identification of a pathogen by a plant occurs through transmembrane pattern recognition receptors (PRRs) or via intracellular proteins of the nucleotide-binding domain (NBD), leucine-rich repeat (NLR) superfamily occurring inside the plant cell [13,14]. Activation of these receptors induces defense reactions, which are highly coordinated with sequence alterations at the cellular level, including the synthesis of signaling molecules such as phytohormones, i.e., salicylic acid (SA), abscisic acid (ABA), jasmonic acid (JA), ethylene (ET) and free radicals arising from hydrogen peroxide (H_2_O_2_) and nitric oxide (NO). Induction of the above-mentioned signaling patterns can cause alterations in gene expression, independently or in dialogue, leading to specific defense responses. Moreover, it has been documented that sugar-induced signal transduction pathways can functionally cooperate with hormonal pathways triggering a complex and extensive signal network in plant cells. These interactions regulate metabolic processes during different phases of plant development as well as during response to stress [15,16,17,18,19,20]. Earlier research has shown the existence of a correlation between sugar-induced signals, ABA and ET during the ontogenetic development of plants [16] as well as between sucrose and SA [21]. Hexose sensing in the secretory pathway was essential for mediating the activation of defense-related genes in tobacco plants with overexpression of the vacuolar and apoplastic invertases [21]. In turn, Loreti et al. [22] point to the existence of mutual reactions between sucrose and phytohormones in the control of anthocyanin biosynthesis in *Arabidopsis thaliana*. Moreover, the results presented by Bogatek et al. [23], pointed to a correlation between JA and sugars. As reported by Tarkowski et al. [24], soluble sugar signaling and dynamics are crucial not only for the control of plant development and organogenesis, but they are also extremely important for coping with biotic and abiotic stresses. According to an extremely interesting concept of “Sweet Immunity” launched by Professor Van den Ende into the international research community, it is postulated that sugar signaling influences plant immune networks and that sugars are essential players in plant defense strategies [2].

The lifestyle of a pathogen may often dictate the host’s defense strategy and the pathogen may even manipulate hormonal crosstalk for successful colonization [25,26]. Many reports reveal that plants’ SA-dependent responses to the attack of pathogenic fungi are considered to be essential in the case of biotrophic pathogens, while JA, ET and ABA are generally associated with plant defense mechanisms against necrotrophic pathogens [27,28,29,30,31,32,33]. In the case of hemibiotrophic pathogens, a transition occurs from biotrophy via a biotrophy-to-necrotrophy switch to necrotrophy, which has been demonstrated by microscopic and transcriptional data [26]. *F. oxysporum* used in these studies is a facultative parasite, i.e., possessing both biotrophic and necrotrophic feeding mechanisms. This pathogen is responsible for Fusarium wilt as well as the pre-emergent sprout root and postemergent seedling root. Additionally, *F. oxysporum* is a systemic pathogen that lives within its host and is able to grow away from the site of infection to other parts of the plant.

The aim of this study was to examine whether the level of saccharides, i.e., sucrose, glucose and fructose, in the embryo axes of yellow lupine (*Lupinus luteus* L. cv. Juno) has an influence on the levels of SA, ABA, ET and H_2_O_2_ as well as on the activity of selected enzymes participating in the biosynthesis of those molecules, i.e., phenylalanine ammonia-lyase (PAL) and benzoic acid 2-hydroxylase (BA2H), engaged in the biosynthesis of SA and the superoxide dismutase (SOD) which is an enzyme catalyzing the reaction of superoxide anion radicals to H_2_O_2_ molecules. Moreover, it was important to demonstrate the correlation between the level of these saccharides and disease symptom development in yellow lupine embryo axes caused by *F. oxysporum* f. sp. *lupini*. Examination of the influence of soluble sugars on the levels of the above-mentioned signaling molecules in yellow lupine in its defense response to hemibiotrophic fungus *F. oxysporum* is completely innovative. It should be emphasized that hemibiotrophic fungi are considered to be the most interesting group of pathogens since they use sequential biotrophic and necrotrophic infection strategies to invade and colonize host plants. The explanation of mixing ratios of the studied signaling molecules in the context of diversified levels of soluble sugars, and also during sugar crosstalk and infection caused by *F. oxysporum* in the embryo axes of *L. luteus*, will contribute to the knowledge of contemporary phytopathology by revealing regulatory mechanisms taking place during host plant–pathogenic fungus interactions. It should be emphasized that, in the present research on the role of sugars in the regulation of the level of endogenous signaling molecules, in vitro cultures of isolated yellow lupine embryo axes were used. After removal of the natural sources of nutrients such as cotyledons, embryo axes of germinating seeds were restricted to accumulate sugars only from the medium. Therefore, through manipulation of the source of organic carbon in the medium, changes in sugar levels in cells and tissues were made.

## 2. Results

### 2.1. Involvement of Soluble Sugars in the Regulation of the Level of Signaling Molecules as Well as in the Enzyme Activity in Embryo Axes of L. luteus L. cv. “Juno” in Response to F. oxysporum

#### 2.1.1. Levels of Salicylic Acid (SA)

**Figure 1:**
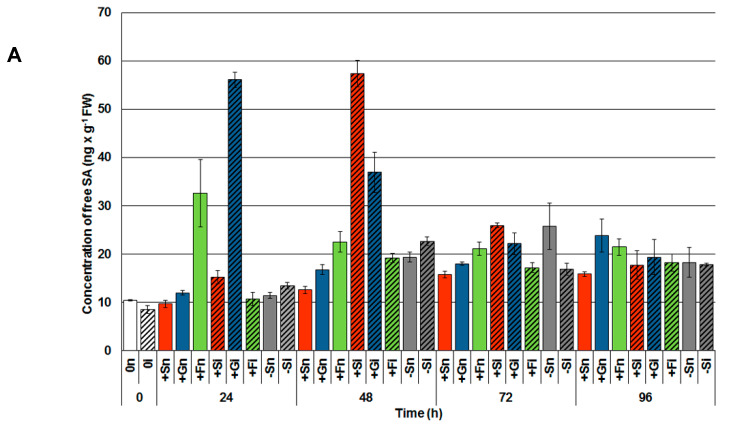

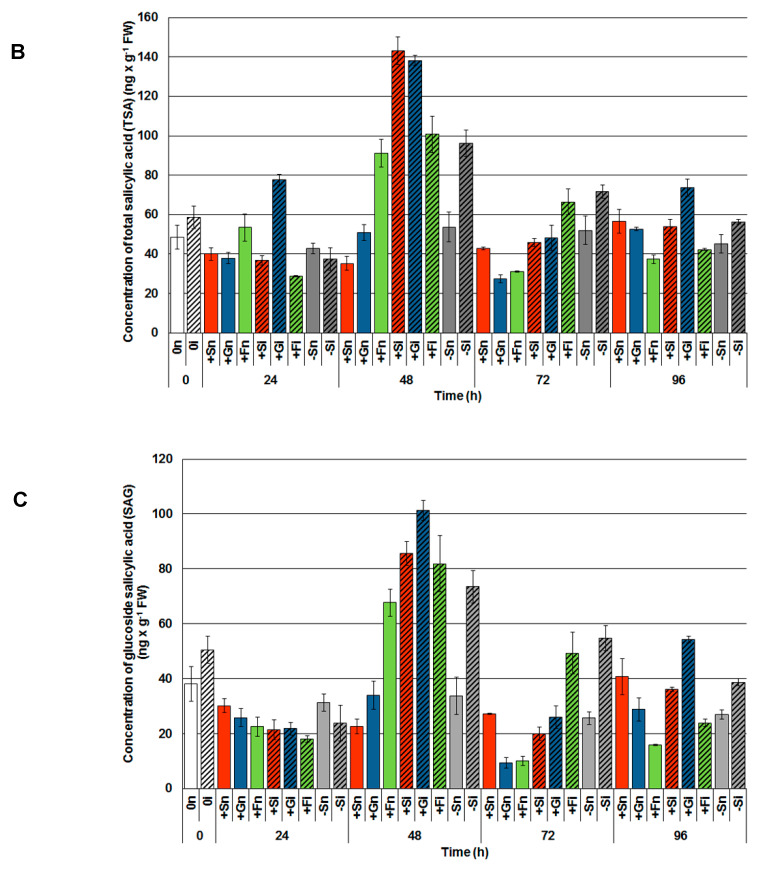
Effect of sucrose, glucose and fructose on the accumulation of free salicylic acid (SA; (**A**)) salicylic acid glucoside (SAG; (**B**)) and total salicylic acid (TSA; (**C**)) in in vitro cultured embryo axes of *Lupinus luteus* infected with *Fusarium oxysporum* f. sp. *lupini*. A summary of the statistical significance of differences between the average values of each pair at *p* < 0.05 can be found in the Supplementary Files (Tables S1–S3).

A very high content of free SA, many times higher than in other experimental variants, was noted already in 24 h inoculated embryo axes cultured on a medium with glucose (Figure 1A); several times higher content of SA than in other experimental variants. A high level of free SA was also noted in noninoculated embryo axes cultured on a medium with fructose (+Fn). However, especially noteworthy is the observation made at 48 h after pathogen inoculation, where the highest content of both free SA (Figure 1A) and its glucoside (salicylic acid glucoside (SAG); Figure 1B) was ascertained in axes inoculated with *F. oxysporum* cultured with sucrose or other monosaccharides; it was considerably higher than in the other experimental variants (Figure 1). The highest content of total salicylic acid (TSA; sum of SA and SAG) was observed at 48 h in the embryo axes inoculated with *F. oxysporum* cultured with sucrose (+Si) or glucose (+Gi; Figure 1C). Additionally, 48 h after inoculation, a high level of SAG was observed in inoculated embryo axes cultured with a sugar deficit (−Si) in comparison to noninoculated ones (−Sn, +Sn or +Gn).

#### 2.1.2. Levels of Abscisic Acid (ABA)

**Figure 2:**
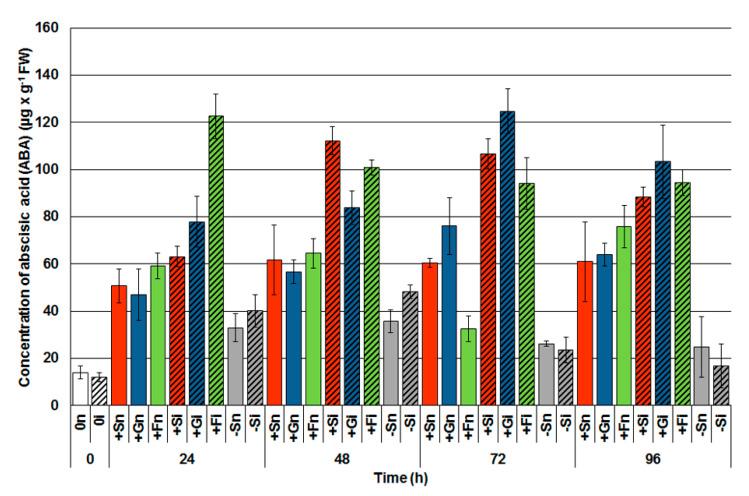
Effect of sucrose, glucose and fructose on the accumulation of abscisic acid (ABA) in in vitro cultured embryo axes of *Lupinus luteus* infected with *Fusarium oxysporum* f. sp. *lupini*. A summary of the statistical significance of differences between the average values of each pair at *p* < 0.05 can be found in the Supplementary Files (Table S4).

It has been shown in Figure 2 that sucrose and monosaccharides (glucose and fructose) alone triggered the accumulation of ABA in the embryo axes of yellow lupine within 24 to 96 h of culturing. The ABA accumulation was significantly higher in noninoculated tissues with a high level of sugars (+Sn, +Gn, +Fn) than in the tissues with a sugar deficit (−Sn). The infection caused a considerable accumulation of ABA in the tissues with a high level of soluble sugars and this situation has been observed at every time points after inoculation. Up to 72 h after inoculation approximately a twice as high ABA level in some of the examined experimental variants with a high endogenous level of sugars (e.g., at 24 h +Fi, 48 h +Si, 72 h +Si and 72 h +Fi embryo axes) in relation to noninoculated axes with a high level of sugars (24 h +Fn, 48 h +Sn, 72 h +Sn and 72 h +Fn, respectively) was observed. It needs to be emphasized that ABA levels in the tissues with a sugar deficit in both uninfected and infected axes were much lower than in tissues with a high level of sugars (Figure 2).

#### 2.1.3. Levels of Ethylene Secretion (ET)

**Figure 3:**
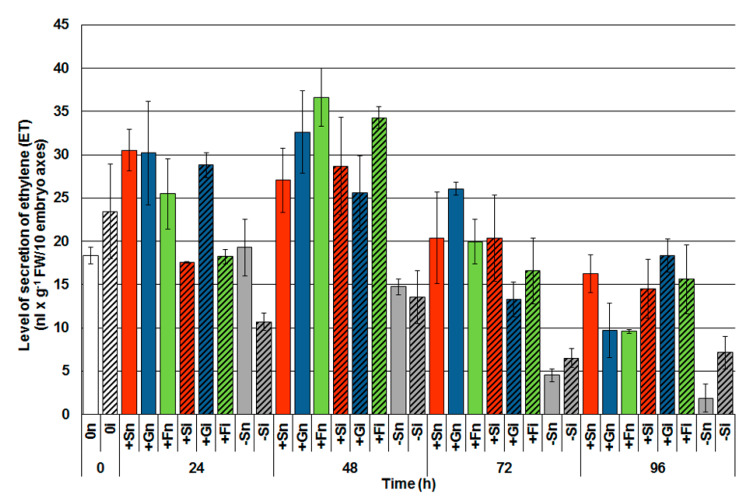
Effect of sucrose, glucose and fructose on the secretion of ethylene (ET) in in vitro cultured embryo axes of *Lupinus luteus* infected with *Fusarium oxysporum* f. sp. *lupini*. A summary of the statistical significance of differences between the average values of each pair at *p* < 0.05 can be found in the Supplementary Files (Table S5).

A headspace method was used for evaluating the secretion of ethylene from embryo axes by applying gas chromatography (GC). Directly after inoculation in embryo axes at 0 h (0i) a higher ET secretion was observed, by 27% in comparison with control axes, i.e., noninoculated (0n; Figure 3). Analysis of the ET secretion within 24 to 96 h of culturing, clearly demonstrated that the soluble sugars alone caused a high ET secretion from embryo axes at every examined time point. The generation of ET was much higher in the tissues with high sugar levels than in the tissues with a sugar deficit. However, up to 72 h after infection, a higher generation of ET in inoculated embryo axes cultivated with high levels of soluble sugars was not observed if compared to the noninoculated ones. On the other hand, in the infected axes with a sugar deficit (−Si) upon the developmental phase of the disease, i.e., at 72 h and 96 h after the inoculation, an increased ET secretion regarding the control tissue (−Sn) was observed. Additionally, 96 h after inoculation, the infected tissues with a high level of monosaccharides (+Gi, +Fi) displayed a higher ET generation than the uninoculated tissues (+Gn, +Fn). Moreover, 96 h after inoculation, a higher ET generation was observed in the infected tissues with a high level of monosaccharides (+Gi, +Fi) than in the noninoculated tissues (+Gn, +Fn).

#### 2.1.4. Hydrogen Peroxide (H_2_O_2_) Concentration

**Figure 4:**
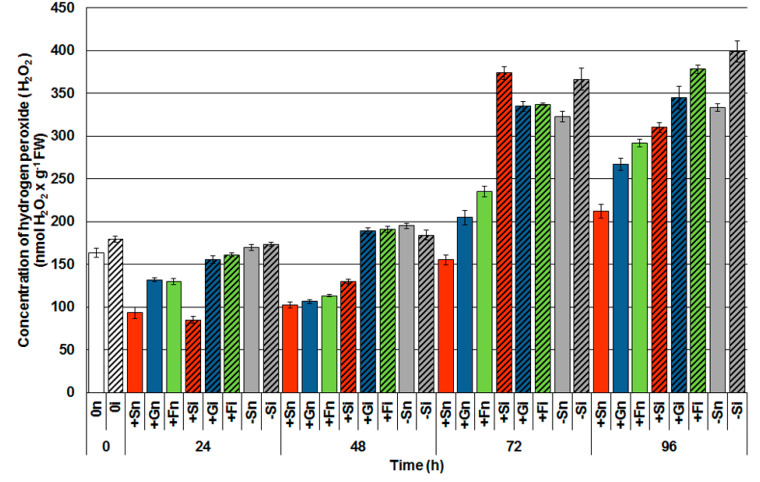
Effect of sucrose, glucose and fructose on the accumulation of hydrogen peroxide (H_2_O_2_) in in vitro cultured embryo axes of *Lupinus luteus* infected with *Fusarium oxysporum* f. sp. *lupini*. A summary of the statistical significance of differences between the average values of each pair at *p* < 0.05 can be found in the Supplementary Files (Table S6).

A spectrophotometric method was used to determine the level of H_2_O_2_. Within 24 to 96 h after inoculation with *F. oxysporum*, a postinfection rise of H_2_O_2_ was observed in inoculated embryo axes with a high level of soluble sugars (Figure 4). Already within 48 h after inoculation, the accumulation of H_2_O_2_ in the axes inoculated with a high level of glucose (+Gi) and fructose (+Fi) was almost twice as high as in noninoculated axes, i.e., +Gn and +Fn. However, a sudden postinfection rise of H_2_O_2_ was observed 72 h and 96 h after inoculation, not only in the tissues with high sugar levels, but also in the tissues with a sugar deficit. It needs to be stressed that increased levels of H_2_O_2_ occurred in embryo axes at late time points after the inoculation. Moreover, 72 h and 96 h following infection, the sugar deficiency alone triggered high H_2_O_2_ generation and it was found to be significantly higher in −Sn tissues than in tissues with high levels of sucrose (+Sn) and monosaccharides (+Gn and +Fn). 

#### 2.1.5. Phenylalanine Ammonia-Lyase (PAL) Activity

**Figure 5:**
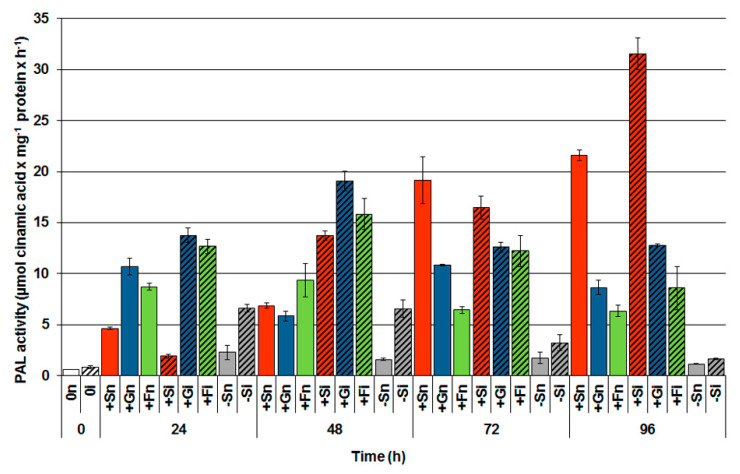
Effect of sucrose, glucose and fructose on the activity of phenylalanine ammonia-lyase (PAL) in in vitro cultured embryo axes of *Lupinus luteus* infected with *Fusarium oxysporum* f. sp. *lupini*. A summary of the statistical significance of differences between the average values of each pair at *p* < 0.05 can be found in the Supplementary Files (Table S7).

Analysis of PAL activity within 0 to 96 h of culturing showed that the activity of this enzyme was considerably higher in the noninoculated axes with a high level of sugars (+Sn, +Gn, +Fn) than in noninoculated axes with a sugar deficit (−Sn; Figure 5). Among soluble sugars, sucrose stimulated PAL activity the strongest in noninoculated embryo axes (+Sn). Additionally, pathogen inoculation significantly increased that activity. The highest activity of PAL was observed 96 h after the inoculation of axes cultured on a medium with sucrose (+Si). PAL activity was also very high in inoculated tissues cultured on a medium with glucose (+Gi) and fructose (+Fi), especially up to 48 h after inoculation and it was much higher than in inoculated tissues with a sugar deficit (−Si).

#### 2.1.6. Benzoic Acid 2-Hydroxylase (BA2H) Activity

**Figure 6:**
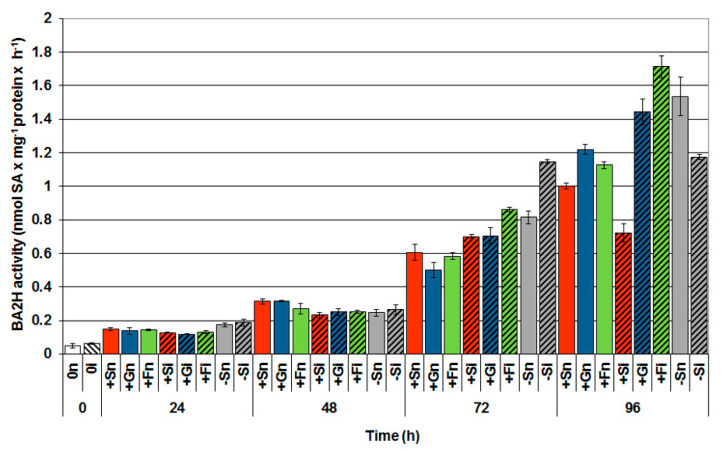
Effect of sucrose, glucose and fructose on the activity of benzoic acid 2-hydroxylase (BA2H) in in vitro cultured embryo axes of *Lupinus luteus* infected with *Fusarium oxysporum* f. sp. *lupini*. A summary of the statistical significance of differences between the average values of each pair at *p* < 0.05 can be found in the Supplementary Files (Table S8).

Between 0 and 24 h of experiment, an increase in BA2H activity in all variants was observed (Figure 6). It was noted that at 48 h of in vitro culture, the activity of this enzyme was considerably higher in the noninoculated axes with a high level of sucrose (+Sn) and monosaccharides (+Gn and +Fn) than in other experimental variants. Moreover, HPLC analysis revealed that infection of embryo axes of yellow lupine enhanced BA2H activity, especially 72 h after inoculation, and continued to rise. At this time point, BA2H activity was higher in infected embryo axes than in noninfected embryo axes. However, the highest enzyme activity was observed in 96 h infected embryo axes cultured on a medium with fructose (+Fi) in comparison to other experimental variants; the activity of BA2H in +Fi variant was approximately 1.5 times higher than in noninoculated embryo axes cultured on the medium with fructose (+Fn) and other sugars (+Sn or +Gn). It should also be stressed that a sugar deficit stimulated BA2H activity in 72 and 96 h noninoculated embryo axes (−Sn).

#### 2.1.7. Superoxide Dismutase (SOD) Activity

**Figure 7:**
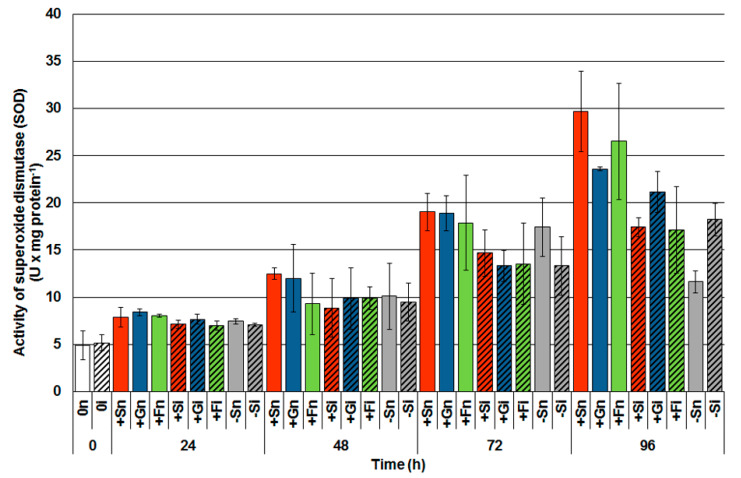
Effect of sucrose, glucose and fructose on the activity of superoxide dismutase (SOD) in in vitro cultured embryo axes of *Lupinus luteus* infected with *Fusarium oxysporum* f. sp. *lupini*. A summary of the statistical significance of differences between the average values of each pair at *p* < 0.05 can be found in the Supplementary Files (Table S9).

The activity of SOD, an enzyme involved in the synthesis of H_2_O_2_ in the infected tissues of *F. oxysporum* with a high level of soluble sugars was lower than in control tissues, i.e., noninoculated with a high level of sugars, 48 h after inoculation (Figure 7). A significant increase in SOD, especially in the noninoculated embryo axes cultured with sucrose (+Sn) and monosaccharides (+Gn and +Fn) was noted after 72 h of culturing. Moreover, at the next time point (96 h), a higher level of SOD in axes inoculated with sugar deficit (−Si) has been observed than in noninoculated (−Sn).

#### 2.1.8. Analysis of Disease Symptoms

Marked differences in the progression of disease symptoms visible on yellow lupine embryo axes inoculated with the systemic acting pathogen, i.e., *F. oxysporum* f. sp. *lupini*, were observed at 72 and 96 h after inoculation (Table 1). Reduced intensity of disease symptoms in embryo axes cultured on a medium with sugar (+Si, +Gi or +Fi) was observed as compared to embryo axes cultured without it (−Si). The emergence of discoloration and a decrease of turgor in inoculated embryo axes were observed. Embryo axes cultured on a medium without sugar (−Si) demonstrated a significant loss of turgor, necrosis and brown discoloration of the whole surface axes and their dieback in comparison to embryo axes cultured on a medium with sugar (+Si, +Gi or +Fi).

## 3. Discussion

Signaling molecules are compounds involved in the regulation of numerous genes, including defense genes triggering a specific metabolic effect. It is well-known that transcription of defense genes induced by pathogens in plants is regulated by a complex signaling network [34,35,36]. In these pathways, signaling molecules such as phytohormones, SA, jasmonates (JA and MeJA), ET, ABA, H_2_O_2_ and NO are identified as secondary signals [37,38,39]. At various times after infection, these signaling molecules can be transiently accumulated and signal transduction induced by these molecules to the cell nucleus causes transcription activation of defense genes [15,16,17].

**Figure 8:**
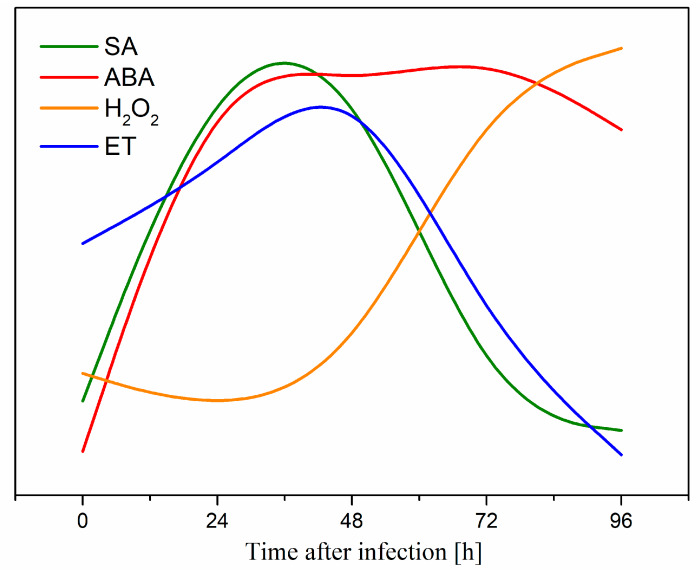
A summary of the dynamics of the accumulation of salicylic acid (SA), abscisic acid (ABA), hydrogen peroxide (H_2_O_2_) and ethylene (ET) molecules in in vitro cultured embryo axes of *Lupinus luteus* infected with *Fusarium oxysporum* f. sp. *lupini*.

This is the first report revealing the involvement of soluble sugars, i.e., sucrose and monosaccharides (glucose and fructose) as primary signals in the regulation of the level of the above-mentioned signaling molecules during the defense response of *L. luteus* to a hemibiotrophic fungus *F. oxysporum* f. sp. *lupini*. There are no data in the literature regarding the effects of sugars on the generation of signaling molecules such as SA, ET, ABA and H_2_O_2_ in the context of an infection caused by pathogenic fungi. It has been shown that the duration of the postinfection generation of these molecules in the case of yellow lupine varied. A summary of the dynamics of the accumulation of these signal molecules is presented in Figure 8. Postinfection generation of these molecules may play a significant role in defense responses of yellow lupine embryo axes [6].

Our earlier research carried out on the same model system has shown an enhanced biosynthesis of isoflavones, particularly free aglycones, generation of superoxide anion radicals (O_2_^•−^) and semiquinone radicals by sucrose, glucose and fructose during the defense response of yellow lupine embryo axes to the hemibiotrophic fungus, *F. oxysporum*. Phytohormones as small organic chemical messengers not only can inhibit or support many developmental processes but also play important roles in plant stress responses through signal mediation or regulation [40,41]. Additionally, it has been documented that the dynamic nature of primary carbon metabolism associated with the effective conversion of sucrose into glucose and fructose by invertases as well as complex interactions of sugar-induced pathways with phytohormone signaling pathways also occurred [1]. Earlier studies also demonstrated the convergence of signal transduction pathways induced by sugars with pathogenic fungus-activated signal transduction pathways [15,42]. Interactions of the above-mentioned signaling pathways also support research by Morkunas et al. [7,9,11].

In recent years, issues regarding sugar metabolism during plant defense response to pathogens and the signal function of these metabolites in a complex interaction network with many phytohormones and reactive oxygen species (ROS) have been raised [24,43]. Both glucose, sucrose as well as fructose are recognized as key regulatory molecules controlling plant defense responses to fungal pathogens [6,11]. It has also been shown that pretreatment with trehalose seeds of susceptible pearl millet provided protection against downy mildew disease [12]. Additionally, spraying with inulin-type fructans or oligogalacturonides reduced gray mold disease symptoms in lettuce and caused accumulation of H_2_O_2_ [24]. During defense response set up, which allows plants to adapt to constantly changing environmental conditions, the role of sugars as signaling molecules that function in a complex signaling network in plant cells becomes more pronounced. This is particularly relevant when discussing their crosstalk with phytohormone-mediated signaling pathways and ROS signaling and other important messengers [44]. The concept regarding the function of sugars as signal molecules inducing priming has just recently been promoted (“sweet priming” or “sweet immunity”). This concept underlines the importance of the role of saccharides in perception, signal transduction and prevention of biotic and abiotic stress [2,45,46]. The results presented here show the correlation between sugar levels and the accumulation of SA and its glucoside SAG (Figure 1A,B) in yellow lupine embryo axes with high carbohydrate levels during the early phase of *F. oxysporum* infection. Over five time higher postinfection accumulation of free SA was recorded in 24 and 48 h embryo axes with a high level of glucose and sucrose (Figure 1A), while SAG accumulation was highest in 48 h infected embryo axes with high sugar levels (Figure 1B). These results may indicate the important role of soluble carbohydrates in SA and SAG levels increase during the biotrophic phase of pathogen development.

There is little information in the literature regarding the effect of sugars on the level of SA [21] and what is more there are no data on the correlation between the carbohydrate content and SA levels in the context of pathogenesis. Herbers et al. [21] showed that treatment of tobacco leaves with sucrose solutions at different concentrations (in the range from 100 to 500 mM sucrose) induced an increase in free and bound SA levels, while this correlation was not found when glucose solutions were used. Among the tested concentrations of sucrose, the highest SA accumulation was observed in the case of 100 mM sucrose. Moreover, many reports in the literature have indicated postinfection elevated SA generation in the host plant [47,48,49,50]. Increased generation of this molecule is generally associated with plant responses to biotrophic and hemibiotrophic pathogens [29,30,33]. SA as an endogenous signaling molecule not only induces local defense responses but, due to the possibility of its transportation by phloem, may participate in the induction of systemically acquired resistance (SAR), which in turn enables the defense against a wide spectrum of pathogens. Fu et al. [51] reported that the NPR1 transcription factor (nonexpresser of PR genes 1) is necessary for SAR activation. Moreover, SA accumulation is often associated with an earlier or parallel increase in the level of PR protein expression [52].

Additionally, this study also demonstrates a strong postinfection increase in PAL activity—an enzyme initiating the phenylpropanoid pathway through which SA can be synthesized—in the embryo axes inoculated with high sugar levels (Figure 5). Furthermore, a high level of PAL activity was also visible in noninoculated embryo axes cultured on the medium with sucrose or monosaccharides; it was considerably higher than in embryo axes cultured with a sugar deficit. We do not exclude that a certain SA pool generated in infected embryo axes with high levels of saccharides can be synthesized not only via the phenylpropanoid pathway, but also via the isochorismate pathway. This is evidenced by the results regarding BA2H activity, where the enzyme activity increased at a later stage of infection and disease, while the generation of free SA was high in the early stages of infection.

It has widely been documented that SA plays an essential role in plant defenses against biotrophic and hemibiotrophic pathogens, while JA, ET and ABA are closely related to resistance to necrotrophic as well as hemibiotrophic pathogens [53,54]. Not only have many recent reports revealed the role of ABA in the induction of resistance to fungal pathogens, but they have also shown its role in the promoting development of infection and disease during plant–pathogen interactions [55,56,57,58]. There is a growing body of literature that reports the mutual dependency of JA, SA and ET in a complex signaling network, in which the different pathways influence each other through positive and negative regulatory interactions [59]. In turn, ABA differently regulates pathogenic responses and is highly dependent on the challenge and the plant species. In addition, it is interesting to note that some saprophytic and parasitic fungi, including *F. oxysporum* may produce ABA [60]. It is likely that pathogens produce plant hormones to modulate hormonal balance in host plant cells thereby affecting defense responses [60]. In this paper, we also provided evidence that apart from a positive correlation between SA and high levels of sugars in tissues, a positive correlation between the content of saccharides and ABA accumulation in the embryo axes of yellow lupine also takes place (Figure 2). It should be emphasized that *F. oxysporum* infection significantly enhances ABA accumulation in tissues with high levels of sugars at every time points, i.e., from 24 h to 96 h after inoculation. Evidently, a very strong generation of ABA in tissues with a high level of sugars may indicate the involvement of this hormone in the defensive responses to *F. oxysporum*. Other authors have also suggested that sucrose may act as a signaling molecule that affects ABA levels and the expression of 9-cis-epoxycarotenoid dioxygenase genes (NCED, 9-cis-epoxycarotenoid dioxygenases), key enzymes regulating ABA biosynthesis [61]. For example, the use of 200 mM sucrose strongly increases the transcript level of NCED. Besides, our results showed that, among three tested sugars (sucrose, glucose and fructose), sucrose treatment led to the highest ABA accumulation in plant tissues. In turn, the results of Cho and Yoo [62] reported positive interactions between fructose signaling and ABA in addition to a negative correlation with ethylene during the early development of *A. thaliana* seedlings. Earlier studies have also shown extensive glucose control over ABA biosynthesis, both via the dependent and nonhexokinase (HXK) routes [16].

The results of our study indicate that ABA as a signaling molecule was strongly generated in the embryo axes of yellow lupine with a high level of saccharides in both the biotrophic as well as necrotrophic *F. oxysporum* development stages (Figure 2). Studies published so far present an increase in ABA levels in the host plant in response to necrotrophic and hemibiotrophic pathogens [49,63,64,65]. There are data supporting that the SA signaling pathway may interact antagonistically with the ABA signal transduction pathway in both NPR1-dependent and independent machineries. These data suggest the existence of many nodal points of antagonism between SA and ABA signaling pathways involved in stress responses [66].

Gas chromatography analyses showed that a high content of soluble carbohydrates caused high secretion of ET from the embryo axes of yellow lupine at all times of culture. However, *F. oxysporum* infection did not increase ET secretion compared to noninoculated axes with high saccharide levels with the exception of the 96 h inoculated embryo axes cultured on the medium with glucose. ET is an important phytohormone that affects most phases of plant ontogenetic development, including seed germination and plant response to biotic stress. The glucose-dependent signaling pathway and probably the mitogen-activated kinase (MAPK) cascade, which are inhibited by ET, have a positive effect on the activation of the common signal transduction pathways affecting the germination process and cotyledon development [67,68]. In addition, Sheen et al. [69] reported that glucose and ET-induced signal pathways were antagonistic to each other. Glucose, whose intracellular sensor is HXK, stimulates glycolysis, the tricarboxylic acid cycle (TCA), and blocks ethylene transduction pathways. The crosstalk between glucose as a signal molecule and the ET-induced pathway has been well proven using, for example, *gin1* (glucose-insensitive) Arabidopsis mutants as reported by Sheen et al. [69]. These authors also indicated that glucose can regulate genes that are controlled by phytohormones such as JA and SA.

H_2_O_2_ is the most studied signal molecule in the context of pathogenesis. In addition, it is known that H_2_O_2_ plays an important role in plant defense responses against biotrophic and necrotrophic pathogens [8,70,71,72,73]. There are a lot of data in the literature on the production of H_2_O_2_ during the defense response of plants against biotrophs and necrotrophs, while results on the interaction between plants and hemibiotrophs are fragmentary. In the present study, we have shown that *F. oxysporum* infection caused an early increased production of H_2_O_2_ in embryo axes with high carbohydrate levels, whereas in carbohydrate-deficient axes, increased H_2_O_2_ content was only observed from 72 h after infection (Figure 4). It should be emphasized that no increase in postinfectional H_2_O_2_ levels was observed in the axes with a high level of carbohydrates compared to axes with a sugar deficit. This result is probably associated with maintaining a high level of O_2_^•−^ generation, which can be included in the defense strategy of the embryo axes [6]. For example, Shetty et al. [74] noted that the infection of wheat with the hemibiotrophic fungus *Septoria tritici* during incompatible interactions resulted in the accumulation of H_2_O_2_. On the other hand, during the compatible interaction, a slight increase was noted in the initial phase of infection. The results obtained in the study of Shetty et al. [74] indicated that H_2_O_2_ is involved in the defense responses of wheat plants during the biotrophic phase of *S. tritici* infection, while the high accumulation of hydrogen peroxide was associated with the necrotrophic growth phase of the pathogen. At the same time, it is interesting to note, in our study, the reduction in SOD activity, an enzyme involved in the scavenging of O_2_^•−^ in infected embryo axes with high carbohydrate levels (Figure 7). This result may be associated with intensive superoxide production in the above tissues vs. time, which may be related to a defense mechanism strategy adopted by these axes against *F. oxysporum* [6]. Our research results also show that embryo axes with high levels of sugars had less development of infection and disease when compared to embryo axes with sugars deficit (Table 1).

## 4. Materials and Methods

### 4.1. Plant Material

Experiments were carried out on yellow lupine (*Lupinus luteus* L. cv. Juno), whose seeds were obtained from the Plant Breeding Company at Tulce near Poznań (Poland). Cultivar ‘Juno’ is resistant to fusariosis according to the data provided by the Plant Breeding Company in Poznań, Poland (the variety was classified to varieties resistant to fusariosis based on breeding tests). Seeds were surface-sterilized, immersed in sterile water and left in an incubator (25 °C). After 6 h of imbibition, the seeds were transferred onto filter paper (in Petri dishes) and immersed in a small amount of water to support further absorption. After a subsequent 18 h, the seed coats were removed from the imbibed seeds and the cotyledons were removed to isolate the embryo axes.

At the beginning of the experiment (time 0 h), the embryo axes were either inoculated with a *Fusarium oxysporum* f.sp. *lupini* spore suspension or they were not inoculated. These were placed, within the next 20 min after cotyledon removal, in groups of four onto Whatman filter papers, which were subsequently transferred to sterile glass test tubes (diameter 3 cm, height 13.5 cm) containing 14 mL of Heller’s mineral medium [75]. There they were suspended in such a way that one end of the axis was immersed in the medium. A space was left below the paper to allow better aeration. After removal of the cotyledons, the embryo axes were dependent on the carbon source provided by the medium. Eight experimental variants were applied: +Sn; embryo axes noninoculated and cultured in vitro on Heller’s medium with 60 mM sucrose, +Gn; embryo axes noninoculated and cultured in vitro on medium with 120 mM glucose; +Fn; embryo axes noninoculated and cultured in vitro on medium with 120 mM fructose; −Sn; noninoculated cultured in vitro on medium without sucrose; +Si; inoculated and cultured with 60 mM sucrose; +Gi; inoculated and cultured with 120 mM glucose; +Fi; inoculated and cultured with 120 mM fructose; −Si; inoculated and cultured without sucrose. It should be mentioned that embryo axes before being transferred to sterile glass test tubes containing Heller’s mineral medium were not inoculated (+Sn, +Gn, +Fn and −Sn) or inoculated with the pathogenic fungus *Fusarium oxysporum* f. sp. *lupini* (+Si, +Gi, +Fi and −Si) as mentioned above. After sugar supply to the medium, the concentrations of endogenous sucrose, glucose and fructose in these embryo axes were previously shown [6]. The applied sucrose, glucose and fructose concentration was optimal to ensure appropriate growth of embryo axes, fresh and dry weight, as well as the uptake of minerals from the medium. Embryo axes were incubated in the dark at 25 °C. Material was weighed, and then was immediately frozen in liquid nitrogen for subsequent analyses of SA and ABA as well as BA2H, and PAL activity. Detection of ET production was performed in fresh materials at particular time points for all variants. Analysis of disease symptoms was performed during the disease phase, i.e., at 72 and 96 h after inoculation.

### 4.2. Preparation of Spore Suspension and Inoculation

*Fusarium oxysporum* f. sp. *lupini* strain K-1018 was obtained from the Collection of Plant Pathogenic Fungi, the Institute of Plant Protection, Poznań. The pathogen was incubated in the dark at 25 °C in Petri dishes (diameter 9 cm) on a potato dextrose agar (PDA) medium (pH 5.5). After 3 weeks of growth an *F. oxysporum* spore suspension was prepared. The spore suspension was obtained by washing the mycelium with sterile water and shaking with glass pearls. Then the number of spores was determined using a hemocytometer chamber (Bürker, Labart, Gdańsk, Poland). Embryo axes were inoculated with the spore suspension at a concentration of 5 × 10^6^ spores per 1 mL. Inoculation was performed by injecting 10 µL of spore suspension into the upper part of the embryo axis shoot and additionally also by spraying the upper part of the embryo axis shoot with the inoculum.

### 4.3. Biochemical Assays

#### 4.3.1. Determination of Salicylic Acid (SA)

Salicylic acid in the free form (SA) as well as that conjugated as a glucoside (SAG) were extracted and quantified following the HPLC method as described [76] with minor modifications. Frozen embryo axes were ground in liquid nitrogen to a fine powder, from which approximately 0.5 g was taken for analysis. SA was extracted twice with methanol 90%, strongly stirred and centrifuged again at 12,000× *g* for 10 min at 4 °C. After centrifugation, the supernatant was divided into two equal parts and the solvent was evaporated to dryness under a stream of nitrogen. A 5% solution of trichloroacetic acid was added to one part and then SA was extracted three times with the extractive organic mixtures of ethyl acetate/cyclopentane/isopropanol (100:99:1, *v*/*v*/*v*). To determine the total (free and glucoside bound) salicylic acid (TSA), 40 units of β-glucosidase in acetate buffer (0.1 M, pH 5.2) were added to the second part of the dry extract and incubated for 90 min at 37 °C. The reaction was terminated by the addition of 5% trichloroacetic acid and then salicylic acid was extracted as described above. After solvent evaporation, the dry residue was dissolved in a mobile phase (0.2 M acetate buffer, pH 5.0; 0.5 mM EDTA) and analyzed by the HPLC method coupled with fluorometric detection with a Waters chromatograph composed of 2699 Separation Module Alliance and 2475 Multi-λ Fluorescence Detector (Waters Corp., Milford, MA, USA). Chromatographic separation was performed on a Spherisorb ODS2 Waters column (3 μm, 4.6 × 10 mm; Waters Corp., Wexford, Ireland). Detection parameters were as follows: 295 nm for excitation and 405 nm for emission. The content of the salicylic acid released from its glucoside (SAG) was calculated as the difference between assays without and with glucoside enzymatic degradation (SAG = TSA − SA) and expressed as nanograms per gram of fresh weight (FW; ng × g^−1^ FW).

#### 4.3.2. Determination of Abscisic Acid (ABA)

The isolation and estimation of abscisic acid (ABA) by HPLC were performed according to Moore [77] with some modifications [78]. Frozen lupine embryo axes (0.5 g) were grounded in liquid nitrogen to a fine powder and homogenized with 80% (*v*/*v*) methanol in 0.2 N acetic acid (3 mL) at 4 °C. ABA-methyl ester was added during homogenization as an internal standard for estimating extraction efficiency. The homogenized tissues were centrifuged at 16,000× *g* for 30 min. The supernatants were collected and the residues were flooded with 1 mL of extraction buffer, stirred and again centrifuged under the above conditions. The combined supernatants (4 mL) were poured into a 15 mL tube and dried in a speed vacuum concentrator. The residues were resuspended in 0.6 mL 80% (*v*/*v*) methanol in 0.2 N acetic acid, taken to 5 mL saturated NaCl solution and partitioned three times against 5 mL diethyl ether. The ether phase was collected, evaporated to dryness and stored at −20 °C until estimation. Dried samples were reconstituted in 150 µL 80% (*v*/*v*) methanol in 0.2 N acetic acid, microfiltered by centrifugation for 10 min at 15,000 × *g*. Samples (20 µL) were injected into a reverse-phase column (Discovery C18, 24 × 4.6 mm, Supelco Inc., Bellefonte, PA, USA), eluted with a gradient of acetonitrile (20–70%) in 0.1% (*v*/*v*) trifluoroacetic acid at the flow rate of 1 mL × min^−1^, and were monitored at 254 nm. Amounts of ABA were calculated from its peak area and the peak area of internal standard using Varian Star 6.3 Chromatography Workstation (Varian Inc., Walnut Creek, CA, USA). The level of ABA was expressed in μg ABA × g^−1^ FW.

#### 4.3.3. Determination of Ethylene (ET)

Measurements of ethylene (ET) generation in lupine embryo axes were performed by the gas chromatography (GC) method [79]. An Agilent 7890A GC instrument with anS/SL injector and a flame ionization detector (FID) was used with a GsBP-PLOT Al_2_O_3_ “KCl” GC column (50 m × 0.32 mm × 8.0 µm; Agilent Technologies Inc., Wilmington, DE, USA). Fresh embryo axes (10 organs) from the control and pathogen fungus-infected were placed in 20 mL glass vials sealed with a Teflon/silicon crimp cap. The volume of 1 mL of air from the vials was injected with a gas-tight syringe into the GC instrument with the isothermal temperature of 110 °C, injector temperature of 140 °C and FID at 240 °C with helium as a carrier gas flowing at 1.3 mL/min. The amount of ET was quantified by comparing the peak height of sample peaks with the standard curve using custom prepared ET in nitrogen of 0.318 ± 0.006 (*v*/*v*; Multax, Zielonki-Parcela, Poland), and was expressed in nanoliters per gram of FW (nL g^−1^ FW).

#### 4.3.4. Determination of Hydrogen Peroxide Concentration

Concentration of hydrogen peroxide (H_2_O_2_) was determined following the spectrophotometric method [80]. Lupine embryo axes (0.5 g) were homogenized with 3 mL of 5% trichloroacetic acid (TCA) and 0.1 g of activated charcoal. The homogenate was filtered through four layers of cheesecloth and centrifuged at 12,000× *g* for 30 min at 4 °C. The reaction mixture contained the extracted solution, 100 mM potassium phosphate buffer (pH 8.4), and a reagent containing 0.6 mM 4-(-2pyridylazo)resorcinol and 0.6 mM potassium-titanium oxalate in 1:1 proportion. The decrease of absorbance (A) was measured at a wavelength of 508 nm in the Perkin Elmer Lambda 15 UV–vis spectrophotometer (Norwalk, CT, USA). Blanks were obtained by using 5% TCA to replace the extracted solution in the mixture. The content of H_2_O_2_ was determined from the difference of A_508_ between each sample and blank. The amount of hydrogen peroxide in lupine embryo axes was expressed as nM H_2_O_2_ × g^−1^ FW.

#### 4.3.5. Phenylalanine Ammonia-Lyase (PAL) Assay

The activity of PAL (EC 4.3.1.24) was determined spectrophotometrically with a modified method of Cahill and McComb [81]. The amount of 0.5 g of frozen lupine embryo axes were homogenized at 4 °C with a mortar and pestle in 4 mL of 0.1 M Tris-HCl buffer (pH 8.9) containing 5 mM mercaptoethanol, and 0.05 g polyvinyl pyrrolidone (PVP). After that, the homogenate was centrifuged at 12,000× *g* for 30 min at 4 °C. The supernatant was used for enzyme analysis. The reaction mixtures contained 0.5 mL of 20 mM borate buffer (pH 8.9), 0.50 mL of 6 mM l-phenylalanine, and 0.1 mL extract and 0.4 mL Tris-HCl buffer (pH 8.9) in a total volume of 1.5 mL. A sample without the substrate l-phenylalanine was used as a blank. The reaction proceeded for 24 h at 30 °C and was terminated by the addition of 1.5 mL of 2 N HCl. PAL activity was measured by the change of absorbance at 290 nm due to the formation of trans-cinnamic acid using a UV–vis spectrophotometer (Norwalk, CT, USA). The activity of PAL is expressed as µM trans-cinnamic acid performed per mg protein per hour (µmol trans-cinnamic acid × mg^−1^ protein × h^−1^).

#### 4.3.6. Benzoic Acid 2-Hydroxylase (BA2H) Assay

The activity of BA2H (EC 1.14.13.-) was measured according to León et al. [82]. The BA2H extraction procedure, enzyme assay and HPLC separation conditions were described in detail in Mai et al. [79]. Briefly, the enzymatic assay mixtures (pH 7.0) consisted of 20 mM 4-(2-hydroxyethyl)-1-piperazineethanesulfonic acid, 1 µM of benzoic acid, 1 µM reduced nicotinamide adenine dinucleotide phosphate and enzyme extract. Samples were incubated for 30 min at 30 °C and next, enzymatic reactions were terminated by 15% trichloroacetic acid addition. The BA2H product, SA was extracted from mixtures with ethyl acetate/cyclopentane/isopropanol (100:99:1, *v*/*v*/*v*) reagent and the number of SA molecules was measured with a Waters Company chromatograph composed of a 2699 Separation Module Alliance and 2475 Multi-λ Fluorescence Detector. The specific activity of BA2H was expressed in moles of formed SA molecules after an hour per mg of protein (nmol SA × mg^−1^ protein × h^−1^).

#### 4.3.7. Superoxide Dismutase (SOD) Assay

The activity of SOD (EC 1.15.1.1) was spectrophotometrically assayed by measuring its ability to inhibit the photochemical reduction of nitroblue tetrazolium (NBT) according to Beauchamp and Fridovich [83]. The frozen lupine embryo axes (0.5 g) were homogenized at 4 °C in 2 mL of 50 mM sodium phosphate buffer (pH 7.0), containing 1.0 mM EDTA, 2% NaCl and 1% PVP and centrifuged at 15,000× *g* for 15 min. The 3 mL reaction mixture contained 50 mM sodium phosphate buffer (pH 7.8), 13 mM methionine, 75 mM NBT, 0.1 mM EDTA and 30 μL of enzyme extract and 2 mM riboflavin (introduced to the reaction mixture as the last reagent). The reaction was started by switching on the light (two 15 W fluorescent lamps placed 30 cm below test tubes) and proceeded for 15 min. Samples without the enzymatic extract in the examined tests were selected so that the absorption difference between blank and examined tests was about 50%. The measurement was carried out with the Perkin Elmer Lambda 15 UV–vis spectrophotometer. The absorbance was recorded at 560 nm. The amount of the enzyme that caused the inhibition of NBT reduction by 50% was taken as a unit of SOD activity. The activity of the enzyme was expressed as units per 1 mg of protein (U × mg^−1^ protein). The protein concentration in enzyme extracts for PAL, BA2H and SOD assays was measured by Bradford’s method [84].

### 4.4. Statistical Analysis

All determinations were conducted within three independent experiments. Additionally, three biological replicates per experimental variant were performed for a given experiment. The normality of the distributions of the studied traits was tested using Shapiro–Wilk’s normality test [85]. The arithmetical means of traits and standard deviations (SDs) were calculated. The elementary comparisons between particular means of physiological–biochemical indices were tested using the two-sample *t*-test, on the 0.05 level. To account for multiple testing, we used the Bonferroni correction. All the analyses were conducted using the GenStat v. 18 statistical software package. A summary of the statistical significance of differences between the average values of each pair of parameters at *p* < 0.05 can be found in the Supplementary Files (Tables S1–S9).

## Figures and Tables

**Table 1:** Disease symptoms of yellow lupine noninoculated embryo axes (n) or inoculated (i) with *Fusarium oxysporum* f. sp. *lupini* and cultured in vitro on Heller’s medium with sucrose (+S), glucose (+G), fructose (+F) or without it (−S).

Culture Variant	Disease Symptoms	
	72 h	96 h
+ Sn	None	None
+ Si	Necrotic changes mainly at the vertices of the shoot axes near the site of inoculation, visible brown discoloration on the roots, slow loss of turgor	Progressing necrosis on the entire surface of the embryo axes, some of the axes without turgor
+ Gn	None	None
+ Gi	Necrotic changes mainly at the vertices of the shoot axes near the site of inoculation, visible brown discoloration on the roots, slightly thicker and longer roots and weaker symptoms in relation to Si + and + Fi, slow loss of turgor	Progressing necrosis on the entire surface of the embryo axes, some of the axes without of turgor, less necrosis compared to + Si and + Fi
+ Fn	None	None
+ Fi	Necrotic changes mainly at the vertices of the shoot axes near the site of inoculation, visible brown discoloration on the roots, slow loss of turgor	Progressing necrosis on the entire surface of the embryo axes, some of the axes without turgor
−Sn	None	none
−Si	Koss of turgor, axes quite substantial overgrow with mycelium, white spots, inhibited elongation growth, brown discoloration almost whole axes, single axes completely white	Significant loss of turgor, axes strongly overgrown with mycelium, necrosis and brown discoloration of whole axes, dieback whole axes, lividity tissues

## Supplementary Materials

Supplementary materials can be found at https://www.mdpi.com/1422-0067/21/11/4133/s1. Table S1. Statistical significance of differences between the average values of each pairs and Student’s t-test for free salicylic acid content, Table S2. Statistical significance of differences between the average values of each pairs and Student’s t-test for glucoside salicylic acid content, Table S3. Statistical significance of differences between the average values of each pairs and Student’s t-test for total salicylic acid content, Table S4. Statistical significance of differences between the average values of each pairs and Student’s t-test for total abscisic acid content, Table S5. Statistical significance of differences between the average values of each pairs and Student’s t-test for ethylene content, Table S6. Statistical significance of differences between the average values of each pairs and Student’s t-test for hydrogen peroxide content, Table S7. Statistical significance of differences between the average values of each pairs and Student’s t-test for phenylalanine ammonia-lyase activity, Table S8. Statistical significance of differences between the average values of each pairs and Student’s t-test for benzoic acid 2-hydroxylase activity. Table S9. Statistical significance of differences between the average values of each pairs and Student’s t-test for superoxide dismutase activity.

## Abbreviations

+Sn	embryo axes noninoculated and cultured in vitro on Heller’s medium with 60 mM sucrose
+Gn	embryo axes noninoculated and cultured in vitro on medium with 120 mM glucose
+Fn	embryo axes noninoculated and cultured in vitro on medium with 120 mM fructose
−Sn	noninoculated cultured in vitro on medium without sucrose
+Si	inoculated and cultured with 60 mM sucrose
+Gi	inoculated and cultured with 120 mM glucose
+Fi	inoculated and cultured with 120 mM fructose
−Si	inoculated and cultured without sucrose
SA	salicylic acid
TSA	total salicylic acid
SAG	glucoside salicylic acid
ABA	abscisic acid
ET	ethylene
PAL	phenylalanine ammonia-lyase
SOD	superoxide dismutase
BA2H	benzoic acid 2-hydroxylase
