# Supplementary material for: The Role of Sugars in the Regulation of the Level of Endogenous Signaling Molecules during Defense Response of Yellow Lupine to Fusarium oxysporum
Source: Int J Mol Sci. 2020 Jun 10;21(11):4133. doi: 10.3390/ijms21114133 (PMC7312090; doi:10.3390/ijms21114133)
Supplement: Supplementary file 1 [file ijms-21-04133-s001.pdf]

Table S1. Statistical significance of differences between the average values of each pairs and Student's t-test for free salicylic acid content. Statistically significant differences (*p*-value) were assumed at $p < 0.05$.

Time	Contrast	Value of contrast	Statistic *t*	*p*	Time	Value of contrast	Statistic *t*	*p*
**0 h**	0n: 0i	1.927	4.05	0.016				
**24 h**	+Sn: +Si	-5.490	-6.22	0.003	**72 h**	-10.19	-20.63	< 0.001
	+Gn:+Gi	-44.05	-46.40	< 0.001		-4.173	-3.19	0.081
	+Fn: +Fi	21.92	5.32	0.006		3.822	3.89	0.018
	-Sn: −Si	-2.037	-3.87	0.018		8.880	3.14	0.035
	+Si: −Si	1.763	2.05	0.110		9.100	11.93	< 0.001
	+Gi: −Si	42.57	42.76	0.001		5.293	3.60	0.023
	+Fi: −Si	-2.780	3.11	0.036		0.3193	0.34	0.748
**48 h**	+Sn: +Si	-44.82	-27.27	< 0.001	**96 h**	-1.867	-1.08	0.388
	+Gn:+Gi	-20.16	-8.08	0.001		4.412	1.54	0.199
	+Fn: +Fi	3.02	2.41	0.073		3.251	2.26	0.086
	-Sn: −Si	-3.273	-4.19	0.014		0.4647	0.26	0.816
	+Si: −Si	34.74	20.84	< 0.001		-0.0930	-0.05	0.962
	+Gi: −Si	14.28	5.77	0.004		1.548	0.74	0.536
	+Fi: −Si	-3.419	-4.56	0.010		0.4037	0.38	0.724

Table S2. Statistical significance of differences between the average values of each pairs and Student's t-test for glucoside salicylic acid content. Statistically significant differences (*p*-value) were assumed at $p < 0.05$.

Time	Contrast	Value of contrast	Statistic *t*	*p*	Time	Value of contrast	Statistic *t*	*p*
**0 h**	0n: 0i	-12.29	-2.68	0.055				
**24 h**	+Sn: +Si	8.694	3.43	0.027	**72 h**	7.200	5.08	0.034
	+Gn:+Gi	4.104	1.74	0.157		-16.82	-6.44	0.003
	+Fn: +Fi	4.534	2.16	0.097		-39.17	-8.75	< 0.001
	-Sn: −Si	7.407	1.78	0.150		-29.07	-9.86	< 0.001
	+Si: −Si	-2.407	-0.56	0.606		-34.75	-11.67	< 0.001
	+Gi: −Si	-2.140	-0.54	0.620		-28.63	-8.11	0.001
	+Fi: −Si	-5.923	-1.55	0.195		-5.478	-1.08	0.342
**48 h**	+Sn: +Si	-63.03	-21.33	< 0.001	**96 h**	4.552	1.21	0.346
	+Gn:+Gi	-67.24	-18.47	< 0.001		-25.32	-9.94	< 0.001
	+Fn: +Fi	-13.39	-2.04	0.110		-8.094	-10.05	< 0.001
	-Sn: −Si	-39.68	-7.69	0.002		-11.60	-9.23	< 0.001
	+Si: −Si	12.18	2.88	0.045		-2.37	-2.65	0.057
	+Gi: -Si	27.78	6.96	0.002		15.70	15.18	< 0.001
	+Fi: −Si	8.369	1.23	0.285		-14.56	-13.26	< 0.001

Table S3. Statistical significance of differences between the average values of each pairs and Student's t-test for total salicylic acid content. Statistically significant differences (*p*-value) were assumed at $p < 0.05$.

Time	Contrast	Value of contrast	Statistic *t*	*p*	Time	Value of contrast	Statistic *t*	*p*
**0 h**	0n: 0i	-10.07	-2.11	0.103				
**24 h**	+Sn: +Si	3.202	1.40	0.235	**72 h**	-3.109	-2.66	0.057
	+Gn:+Gi	-39.88	-17.35	< 0.001		-21.03	-5.46	0.005
	+Fn: +Fi	24.79	6.21	0.025		-35.35	-9.41	0.011
	-Sn: −Si	5.407	1.47	0.215		-19.59	-4.25	0.013
	+Si: −Si	-0.6410	-0.18	0.868		-25.65	-11.46	< 0.001
	+Gi: -Si	40.33	10.82	< 0.001		-23.31	-5.61	0.005
	+Fi: −Si	-8.703	-2.58	0.122		-5.157	-1.22	0.289
**48 h**	+Sn: +Si	-107.9	-24.04	< 0.001	**96 h**	2.684	0.66	0.544
	+Gn:+Gi	-87.40	-31.15	< 0.001		-20.91	-8.03	0.001
	+Fn: +Fi	-9.723	-1.44	0.223		-4.961	-3.90	0.018
	-Sn: −Si	-42.99	-7.28	0.002		-11.13	-4.03	0.016
	+Si: −Si	46.92	8.36	0.001		-2.498	-1.13	0.323
	+Gi: −Si	42.07	10.03	< 0.001		17.25	6.58	0.003
	+Fi: −Si	4.615	0.70	0.524		-14.15	-20.81	0.001

Table S4. Statistical significance of differences between the average values of each pairs and Student's t-test for total abscisic acid content. Statistically significant differences (*p*-value) were assumed at $p < 0.05$.

Time	Contrast	Value of contrast	Statistic *t*	*p*	Time	Value of contrast	Statistic *t*	*p*
**0 h**	0n: 0i	1.990	1.01	0.371				
**24 h**	+Sn: +Si	-2.947	-0.61	0.576	**72 h**	-46.36	-11.98	< 0.001
	+Gn:+Gi	-31.03	-3.53	0.024		-48.66	-5.53	0.005
	+Fn: +Fi	-63.48	-10.02	< 0.001		-61.72	-8.74	< 0.001
	-Sn: −Si	-7.29	-1.40	0.235		2.707	0.85	0.445
	+Si: −Si	13.50	2.89	0.045		83.29	17.19	< 0.001
	+Gi: −Si	37.76	5.16	0.007		101.2	16.09	< 0.001
	+Fi: −Si	82.40	12.17	0.001		70.75	10.07	< 0.001
**48 h**	+Sn: +Si	-50.46	-5.47	0.005	**96 h**	-27.38	-2.71	0.054
	+Gn:+Gi	-27.16	-5.45	0.006		-39.24	-4.16	0.014
	+Fn: +Fi	-36.42	-9.12	< 0.001		-18.61	-3.05	0.038
	-Sn: −Si	-12.60	-3.92	0.017		8.033	0.88	0.429
	+Si: −Si	63.92	17.00	< 0.001		71.55	11.97	< 0.001
	+Gi: −Si	35.56	8.18	0.001		86.32	8.21	0.001
	+Fi: −Si	52.58	21.26	< 0.001		77.63	12.35	< 0.001

Table S5. Statistical significance of differences between the average values of each pairs and Student's t-test for ethylene content. Statistically significant differences (*p*-value) were assumed at p < 0.05.

Time	Contrast	Value of contrast	Statistic *t*	*p*	Time	Value of contrast	Statistic *t*	*p*
**0 h**	0n: 0i	-5.090	-1.59	0.186				
**24 h**	+Sn: +Si	12.96	9.33	0.011		0.0267	0.01	0.995
	+Gn:+Gi	1.410	0.040	0.712		12.73	10.27	< 0.001
	+Fn: +Fi	7.260	3.03	0.039		3.380	1.27	0.274
	-Sn: −Si	8.580	4.34	0.012	**72 h**	-1.963	-2.57	0.062
	+Si: −Si	6.857	11.75	0.007		13.88	4.71	0.009
	+Gi: −Si	18.09	17.97	< 0.001		6.827	5.16	0.007
	+Fi: −Si	7.543	10.05	< 0.001		10.09	4.40	0.012
	+Sn: +Si	-1.643	-0.42	0.695		1.790	0.76	0.488
	+Gn:+Gi	7.053	1.89	0.132		-8.687	-4.06	0.015
	+Fn: +Fi	2.383	1.15	0.315		-6.040	-2.62	0.119
**48 h**	-Sn: −Si	1.180	0.64	0.558	**96 h**	-5.253	-3.70	0.021
	+Si: −Si	15.14	4.08	0.015		7.373	3.28	0.031
	+Gi: −Si	12.02	3.91	0.017		11.26	7.31	0.002
	+Fi: −Si	20.71	10.84	< 0.001		8.497	3.35	0.029

Table S6. Statistical significance of differences between the average values of each pairs and Student's t-test for hydrogen peroxide content. Statistically significant differences (*p*-value) were assumed at p < 0.05.

Time	Contrast	Value of contrast	Statistic *t*	*p*	Time	Value of contrast	Statistic *t*	*p*
**0 h**	0n: 0i	-15.96	-4.29	0.013				
**24 h**	+Sn: +Si	8.190	1.83	0.142		-218.5	-38.52	< 0.001
	+Gn:+Gi	-23.96	-8.53	0.001		-130.2	-22.48	< 0.001
	+Fn: +Fi	-31.00	-12.17	< 0.001		-102.1	-28.07	< 0.001
	-Sn: −Si	-3.593	-1.52	0.203	**72 h**	-43.69	-5.31	0.006
	+Si: −Si	-88.53	-36.92	< 0.001		7.350	0.85	0.443
	+Gi: −Si	-17.69	-6.31	0.003		-31.45	-3.89	0.018
	+Fi: −Si	-12.69	-7.64	0.002		-29.23	-3.93	0.057
	+Sn: +Si	-27.72	-10.84	< 0.001		-97.67	-17.37	< 0.001
	+Gn:+Gi	-82.41	-31.78	< 0.001		-77.41	-8.87	< 0.001
	+Fn: +Fi	-77.45	-34.05	< 0.001		-86.58	-22.66	< 0.001
**48 h**	-Sn: −Si	11.10	2.95	0.042	**96 h**	-65.80	-8.74	< 0.001
	+Si: −Si	-54.09	-15.10	< 0.001		-88.88	-11.25	< 0.001
	+Gi: −Si	4.880	1.25	0.281		-54.25	-5.18	0.007
	+Fi: −Si	6.617	1.69	0.166		-20.71	-2.71	0.054

Table S7. Statistical significance of differences between the average values of each pairs and Student's t-test for phenylalanine ammonia-lyase activity. Statistically significant differences (*p*-value) were assumed at $p < 0.05$.

Time	Contrast	Value of contrast	Statistic *t*	*p*	Time	Value of contrast	Statistic *t*	*p*
**0 h**	0n: 0i	-0.2500	-3.02	0.039				
**24 h**	+Sn: +Si	2.897	22.23	< 0.001		2.680	1.84	0.140
	+Gn:+Gi	-3.067	-4.78	0.009		-1.713	-5.97	0.004
	+Fn: +Fi	-3.943	-8.88	< 0.001		-5.783	-6.40	0.003
	−Sn: −Si	-4.293	-9.49	< 0.001	**72 h**	-1.477	-2.70	0.054
	+Si: −Si	-4.847	-20.66	< 0.001		13.25	16.90	< 0.001
	+Gi: −Si	7.183	15.26	< 0.001		9.350	16.90	< 0.001
	+Fi: −Si	6.073	13.43	< 0.001		9.003	9.09	< 0.001
	+Sn: +Si	6.900	-23.17	< 0.001		-9.930	-10.42	< 0.001
	+Gn:+Gi	-13.23	-21.24	< 0.001		-4.150	-10.11	0.008
	+Fn: +Fi	-6.480	-5.02	0.007		-2.237	-1.78	0.149
**48 h**	-Sn: −Si	-4.973	-9.92	0.009	**96 h**	-0.4930	-8.94	< 0.001
	+Si: −Si	7.187	12.85	< 0.001		29.89	32.93	< 0.001
	+Gi: −Si	12.53	16.82	< 0.001		11.15	144.97	< 0.001
	+Fi: −Si	9.270	9.19	< 0.001		6.948	5.71	0.029

Table S8. Statistical significance of differences between the average values of each pairs and Student's t-test for benzoic acid 2-hydroxylase activity. Statistically significant differences (*p*-value) were assumed at $p < 0.05$.

Time	Contrast	Value of contrast	Statistic *t*	*p*	Time	Value of contrast	Statistic *t*	*p*
**0 h**	0n: 0i	-0.01401	-1.75	0.156				
**24 h**	+Sn: +Si	0.02437	4.03	0.016		-0.08344	-2.98	0.041
	+Gn:+Gi	0.01978	1.66	0.172		-0.2048	-5.45	0.006
	+Fn: +Fi	0.01260	2.07	0.107		-0.2779	-21.59	< 0.001
	-Sn: -Si	-0.01213	-0.96	0.391	**72 h**	-0.3328	-14.45	< 0.001
	+Si: −Si	-0.06311	-5.88	0.025		-0.4574	-52.89	< 0.001
	+Gi: -Si	-0.06801	-6.30	0.030		-0.4421	-15.15	< 0.001
	+Fi: −Si	-0.05652	-4.76	0.009		-0.2848	-29.74	< 0.001
	+Sn: +Si	0.08134	6.29	0.003		0.2797	8.31	0.001
	+Gn:+Gi	0.06576	5.18	0.007		-0.2271	-4.82	0.009
	+Fn: +Fi	0.01886	1.01	0.370		-0.5867	-15.26	< 0.001
**48 h**	-Sn: −Si	-0.01778	-0.87	0.435	**96 h**	0.3612	5.39	0.030
	+Si: −Si	-0.03097	-1.63	0.179		-0.4546	-13.55	< 0.001
	+Gi: −Si	-0.1291	-0.62	0.567		0.2696	6.00	0.004
	+Fi: −Si	-0.01197	-0.68	0.531		0.5732	14.09	< 0.001

Table S9. Statistical significance of differences between the average values of each pairs and Student's t-test for superoxide dismutase activity. Statistically significant differences (*p*-value) were assumed at $p < 0.05$.

Time	Contrast	Value of contrast	Statistic *t*	*p*	Time	Value of contrast	Statistic *t*	*p*
**0 h**	0n: 0i	-0.2756	-0.31	0.766				
**24 h**	+Sn: +Si	0.7278	1.27	0.251	**72 h**	4.362	2.75	0.033
	+Gn:+Gi	0.7415	2.47	0.049		5.617	4.59	0.004
	+Fn: +Fi	1.005	3.90	0.008		4.350	1.32	0.235
	-Sn: −Si	0.3569	2.08	0.083		4.161	1.89	0.107
	+Si: −Si	0.0652	0.27	0.793		1.420	0.71	0.501
	+Gi: −Si	0.5912	2.26	0.065		0.0403	0.02	0.982
	+Fi: −Si	-0.0552	-0.22	0.835		0.2741	0.10	0.921
**48 h**	+Sn: +Si	3.614	2.30	0.099	**96 h**	12.26	5.57	0.009
	+Gn:+Gi	2.119	0.87	0.416		2.449	2.24	0.110
	+Fn: +Fi	0.3018	0.17	0.868		9.412	2.45	0.050
	-Sn: −Si	0.6199	0.31	0.770		-6.583	-6.40	< 0.001
	+Si: −Si	-0.5996	-0.33	0.755		-0.7844	-0.79	0.458
	+Gi: −Si	0.4106	0.21	0.837		2.915	2.11	0.080
	+Fi: −Si	-0.4888	-0.42	0.690		-1.089	-0.44	0.672
